# Dose-Responses Relationship in Glucose Lowering and Gut Dysbiosis to Saskatoon Berry Powder Supplementation in High Fat-High Sucrose Diet-Induced Insulin Resistant Mice

**DOI:** 10.3390/microorganisms9081553

**Published:** 2021-07-21

**Authors:** Ruozhi Zhao, Fei Huang, Garry X. Shen

**Affiliations:** 1 Department of Internal Medicine, University of Manitoba, Winnipeg, MB R3E 3P4, Canada; Ruozhi.Zhao@umanitoba.ca (R.Z.); huangf1@myumnitoba.ca (F.H.); 2 Department of Food and Human Nutritional Sciences, University of Manitoba, Winnipeg, MB R3E 3P4, Canada

**Keywords:** Saskatoon berry, dose-response, mice, high fat-high sucrose diet, fasting plasma glucose, inflammation, gut microbiota

## Abstract

Administration of freeze-dried powder of Saskatoon berry (SB), a popular fruit enriched with antioxidants, reduced glucose level, inflammatory markers and gut microbiota disorder in high fat-high sucrose (HFHS) diet-induced insulin resistant mice. The present study examined the dose-response relationship in metabolic, inflammatory and gut microbiotic variables to SB power (SBp) supplementation in HFHS diet-fed mice. Male C57 BL/6J mice were fed with HFHS diet supplemented with 0, 1%, 2.5% or 5% SBp for 11 weeks. HFHS diet significantly increased the levels of fast plasma glucose (FPG), cholesterol, triglycerides, insulin, homeostatic model assessment of insulin resistance (HOMA-IR), tumor necrosis factor-α, monocyte chemotactic protein-1 and plasminogen activator inhibitor-1, but decreased fecal *Bacteroidetes* phylum bacteria and *Muribaculaceae* family bacteria compared to low fat diet. SBp dose-dependently reduced metabolic and inflammatory variables and gut dysbiosis in mice compared with mice receiving HFHS diet alone. Significant attenuation of HFHS diet-induced biochemical disorders were detected in mice receiving ≥1% SBp. The abundances of *Muribaculaceae* family bacteria negatively correlated with body weights, FPG, lipids, insulin, HOMA-IR and inflammatory markers in the mice. The results suggest that SBp supplementation dose-dependently attenuated HFHS diet-induced metabolic and inflammatory disorders, which was associated with the amelioration of gut dysbiosis in the mice.

## 1. Introduction

The prevalence of diabetes has rapidly increased worldwide. The primary type of diabetes contributing to this increase is type 2 diabetes (T2D) [1]. T2D is characterized by insulin resistance and is often associated with obesity [2]. Although most T2D patients are middle- or old-aged people, increasingly, cases of T2D have been detected in youths [3]. Unhealthy diet, lack of physical activity and genetic factors contribute to the epidemic of T2D [4]; diets containing high level of fat and/or sugar play an important role in the development of obesity, insulin resistance and T2D [5]. T2D is associated with low-grade chronic inflammation [6]. Gut microbiota is regulated by daily diet and implicated in both metabolism and inflammation [7]; accumulating lines of evidence suggest that gut dysbiosis contributes to the development of T2D and obesity [8]. 

Saskatoon berry (SB, *Amelanchier alnifolia Nutt.*) is a type of high shrub that naturally grows in Canada and some northern states of the USA [9], and was more recently planted in Europe [10]. The fruits of SB have a taste of light sweet and high levels of phenolic antioxidants [11]; the content of total anthocyanins in dried SB was found to be 1.5-fold, 3.17-fold and 5.77-fold of that in raspberry, chokecherry and strawberry, respectively, and similar to that in blueberry (1.01-fold) [12]. 

A previous study by our group demonstrated that supplementation of 5% SB powder (SBp) in high fat-high sucrose (HFHS) diet significantly lowered fasting plasma glucose (FPG) and insulin resistance in mice, which was associated with reductions in inflammatory markers and gut dysbiosis [13]. Our findings regarding the glucose-lowering effect of SBp was supported by a separate group of researchers, in whose experiment rats were fed with high fat and high carbohydrate diet supplemented with 2.7% SBp [14]. The dose-response to SBp administration on glucose lowering, insulin resistance, inflammatory markers and gut microbiota in animal models remains unclear.

The present study examines the effects of supplementation with 1–5% SBp in HFHS diet on FPG, lipids, insulin resistance, inflammatory markers and gut microbiota in a diet-sensitive rodent model, C57 BL/6J mice, compared with mice fed HFHS diet alone. Relationships between multiple SBp doses, and changes of metabolic, inflammatory and gut microbiotic variables in the mice were investigated.

## 2. Materials and Methods

### 2.1. Animal Model

Male C57 BL/6J mice (*n* = 40, 6 weeks of age) were received from the Jackson Laboratory (Bar Harbor, ME, USA). Mice were housed in an air-conditioned room with an alternating 12 h day/night light cycle and received regular mouse chow and tap water for 1 week to stabilize. The protocols of animal experiment have been approved by the Animal Care Committee at the University of Manitoba.

### 2.2. Dietary Regimens

Smoky Saskatoon berries obtained from the Prairie Lane Saskatoon (Portage, MB, Canada). SBp was prepared via lyophilization of frozen Saskatoon berry and stored at −80 °C [15]. Mice were randomized into five groups (*n* = 8/group, hosted *n* = 4/cage) and received one of following the diets for 11 weeks: (1) control group, receiving D12450K low fat diet from Research Diets (New Brunswick, NJ, USA) containing 4.3% fat, 19.2% protein, 67.3% carbohydrates (mass/mass) without an addition of sucrose; (2) HFHS group fed with HFHS diet (D12492, Research Diets) containing 35% fat, 26% protein and 26% carbohydrates, including 9% sucrose; (3) 1% SBp group fed HFHS diet supplemented with 1% SBp (mass/mass); (4) 2.5% SBp group fed HFHS diet supplemented with 2.5% SBp; (5) 5% SBp group fed HFHS diet supplemented with 5% SBp.

### 2.3. Animal Monitoring and Sample Collection

Body weights and food intake of animals were assessed at onset and every other week for 10 weeks from beginning each dietary experiment. Blood was collected from mouse saphenous veins at onset and every other week after an overnight fasting (14 h) to measure levels of plasma glucose and thus monitor any development of diabetes. Mice were euthanized at the 11th week after the onset of the dietary experiment via the inhalation of 5% isoflurane, minimizing the animals’ pain. Blood was withdrawn subsequently by heart puncture.

### 2.4. Analyses of Metabolic Variables 

The levels of glucose and cholesterol in the fasting plasma of mice were analyzed using Sekisui Diagnostics SL reagent kits (Charlottetown, PE, USA). Plasma levels of triglycerides were measured using BioAssay reagents (Hayward, CA, USA). Insulin levels in plasma were assessed using enzyme-linked immunosorbent assay (ELISA) kits from EMD Millipore (Billerica, MA, USA) for mouse insulin. The homeostatic model assessment of insulin resistance (HOMA-IR) of the mice was calculated from plasma insulin and glucose from simultaneously withdrawn blood samples, as previously described [16]. 

### 2.5. Measurement of Circulating Inflammatory Markers

The levels of tumor necrosis factor-α (TNFα), monocyte chemotactic protein-1 (MCP-1) and plasminogen activator inhibitor-1 (PAI-1) in plasma were measured using ELISA kits from Bioscience (San Diego, CA, USA) for mouse TNFα, from Thermo Fisher Scientific (Ottawa, ON, USA), for mouse MCP-1 and for mouse PAI-1 from Oxford Biomedical Research (Oxford, MI, USA), respectively [13]. 

### 2.6. Fecal Sample Collection 

Mice were housed in singly hosted cages with fresh bedding overnight for one night during the 10th week after the start of the dietary experiment and returned to normal host condition afterwards. Fecal pellets were collected from individually housed mice, and stored fecal samples in separated tubes at −80 °C before further analysis.

### 2.7. Fecal Bacteria DNA Extraction and Sequencing

Fecal DNA was extracted using PowerFecal DNA Isolation Kit (QIAGEN, Germantown, MD, USA) and quantified using a NanoDrop 2000 spectrophotometer (Thermo Scientific). Bacteria DNA in mouse feces was amplified using polymerase chain reaction (PCR) with primers containing 515F (5′-GTGYCAGCMGCCGCGGTAA) and 926R (5′-CCGYCAATTYMTTTRAGTTT) targeting the V4-V5 region of bacterial DNA. A high throughput Hamilton Nimbus Select robot and Coastal Genomics analytical gels were run to verify the quality of PCR products. The PCR amplicons were normalized by using a high throughput Charm Biotech Just-a-Plate 96-well normalization kit, then pooled to construct a library and quantified before sequencing on an Illumina MiSeq platform in the Integrated Microbiome Resource at Dalhousie University [17].

### 2.8. Bioinformatics Analysis and Statistics 

Raw data of gut microbiota in the form of fastq files was demultiplexed according to barcode sequences, followed by trimming using Cutadapt (version 1.17) to remove primers. Trimmed reads were imported as artifact into an open-source bioinformatics pipeline of decentralized microbiome analysis package of Quantitative Insights Into Microbial Ecology 2 (QIIME2, version: 2018. 8) [18]. Taxonomies were assigned to amplicon sequence variants using a Naive-Bayes approach and SILVA database. Diversity metrics (Core-metrics-phylogenetic) within QIIME2 were used to evaluate α- and β-diversity of gut microbiota. Differences between data from multiple groups were examined using the analysis of variance (ANOVA) and post-hoc Tukey test. Significant difference was preset at *p* < 0.05 as previously described [19]. 

## 3. Results

### 3.1. Impact of HFHS Diet on Body Weights, Metabolic and Inflammatory Variables

HFHS diet significantly increased body weights, FPG, cholesterol, triglycerides, insulin, HOMA-IR and inflammatory markers (PAI-1, TNFα and MCP-1) in mice compared with low fat diet after 11 weeks of dietary intervention (*p* < 0.01, Table 1). No significant difference was detected in the mass of food intake between groups of mice receiving low fat and HFHS diet (data not shown).

### 3.2. Effects of SBp Supplementation on HFHS Diet-Induced Metabolic and Inflammatory Variables and Body Weight in Mice

**Figure 1:**
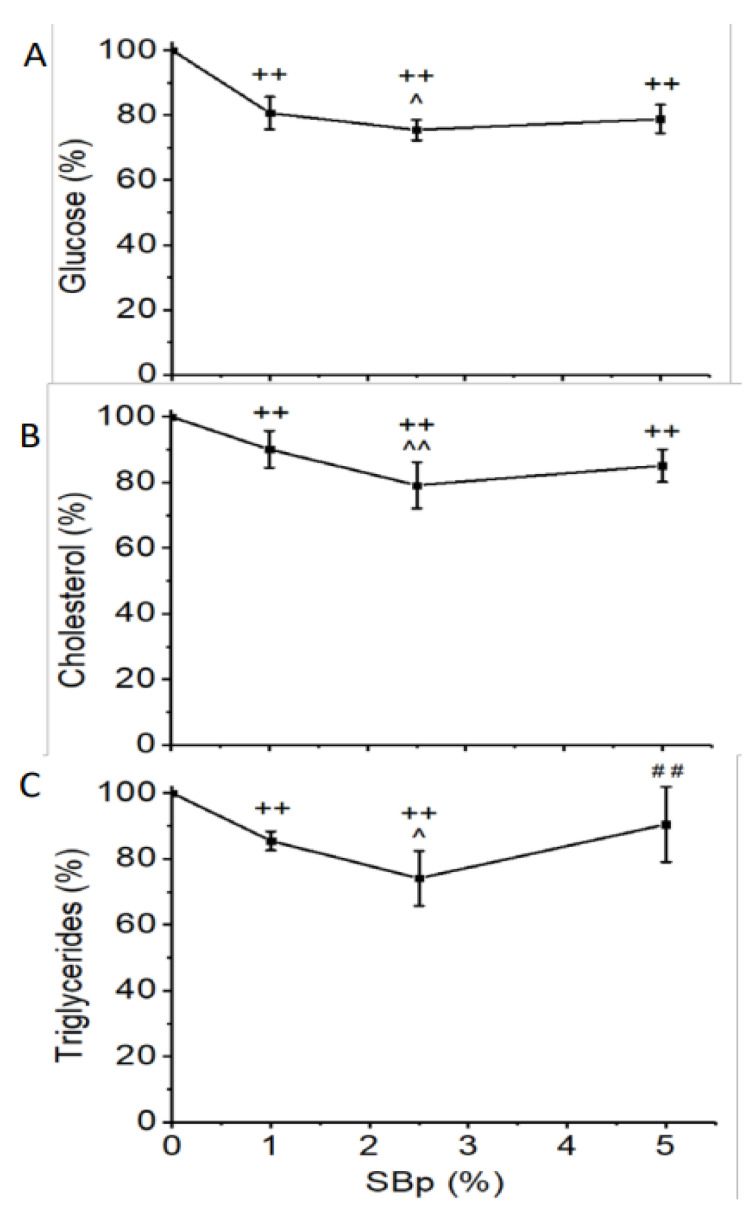
Levels of glucose, cholesterol and triglycerides in plasma of male C57 BL/J6 mice fed with HFHS diets supplemented with 0–5% Saskatoon berry powder (SBp). Male C57 BL/J6 mice (6 weeks of age) were randomized into four groups and received following diets for 11 weeks: HFHS group: HFHS diet without addition of SBp; 1% SBp group: 1% SBp supplemented HFHS diet; 2.5% SBp group: 2.5% SBp supplemented HFHS diet; and 5% SBp group: 5% SBp supplemented HFHS diet. Fasting plasma glucose, cholesterol and triglycerides were measured in blood samples collected at the end of the intervention. Relative changes of fasting plasma glucose (**A**), total cholesterol (**B**) and triglycerides (**C**) vs. HFHS diet group were expressed in mean ± SD % (*n* = 8/group). ++: *p* < 0.01 vs. the HFHS group; ^, ^^: *p* < 0.05 or 0.01 vs. 1% SBp group; ##: *p* < 0.01 vs. 2.5% group.

Supplementation with SBp 1–5% significantly decreased FPG, cholesterol and triglycerides in plasma after overnight fasting compared with HFHS diet alone (*p* < 0.01). The reduction of FPG or lipids in mice treated with 2.5% SBp was significantly lower than that in mice receiving 1% SBp (*p* < 0.05 or 0.01). No significant difference in FPG or lipids between mice treated with HFHS + 5% SBp and mice receiving HFHS + 1% or those fed with HFHS + 2.5% SBp diet (Figure 1A–C) were found.

**Figure 2:**
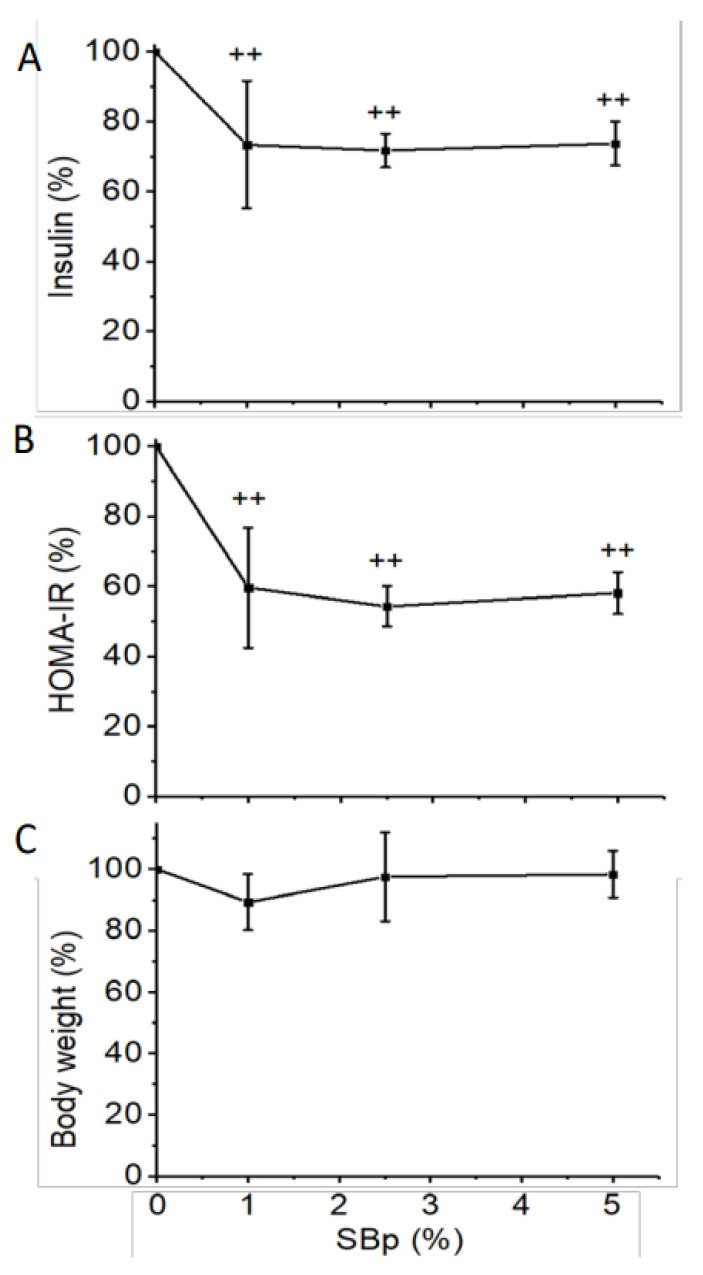
Effects of HFHS diets supplemented with 0–5% SBp on insulin, insulin resistance and body weight in mice. The experimental regimen was described in the legend of Figure 1. Homeostatic model assessment of insulin resistance (HOMA-IR) measurements were analyzed from the levels of insulin and glucose in fasting plasma collected at the end of the intervention. Body weights were measured at the last weeks of the intervention. Relative changes in insulin (**A**), HOMA-IR (**B**) or body weights (**C**) vs. HFHS group were expressed in mean ± SD % (*n* = 8/group). ++: *p* < 0.01 vs. HFHS group.

Supplementation with 1–5% SBp significantly decreased the relative plasma levels of insulin and HOMA-IR in mice compared to HFHS diet alone. No significant difference in insulin or HOMA-IR was detected between mice treated with different dosages of SBp (Figure 2A,B). Supplementation of SBp in HFHS diet did not significantly alter the relative changes in body weights of mice compared to that in mice receiving HFHS diet alone (Figure 2C). 

**Figure 3:**
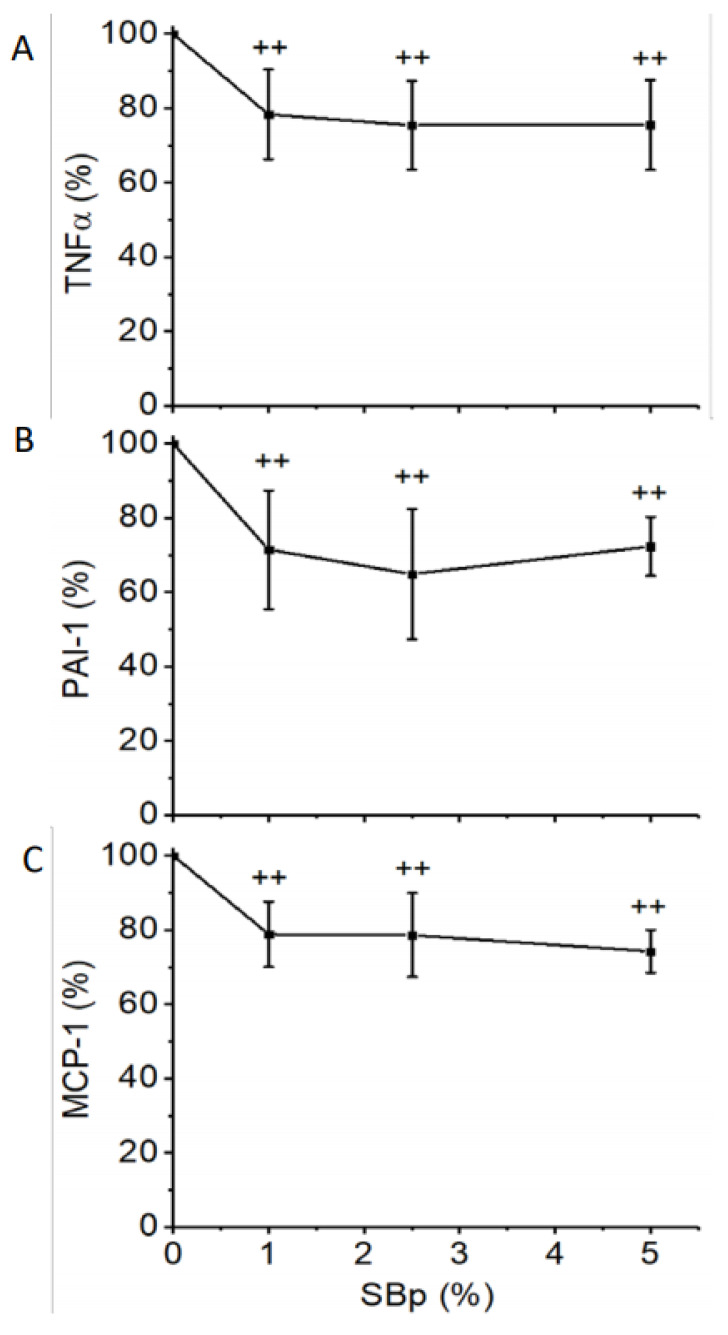
Levels of inflammatory regulators in plasma of mice receiving HFHS diet supplemented with 0–5% SBp. The experimental regimen is described in the legend of Figure 1. Tumor necrosis factor-α (TNFα in **A**), plasminogen activator inhibitor-1 (PAI-1 in **B**) and monocyte chemotactic protein-1 (MCP-1 in **C**) were analyzed in plasma collected at the end of the intervention. Relative changes in the inflammatory markers were expressed in mean ± SD % (*n* = 8/group) vs. HFHS group. ++: *p* < 0.01 vs. the HFHS group.

The relative plasma levels of TNFα, PAI-1 and MCP-1 in mice treated with HFHS diet supplemented with 1, 2.5 or 5% SBp were significantly lower than mice treated with HFHS diet alone. No significant difference in the inflammatory markers was detected between mice treated with different dosages of SBp (Figure 3A–C). 

### 3.3. Dose-Dependence to SBp on the Diversities of Gut Microbiota in Mice 

**Figure 4:**
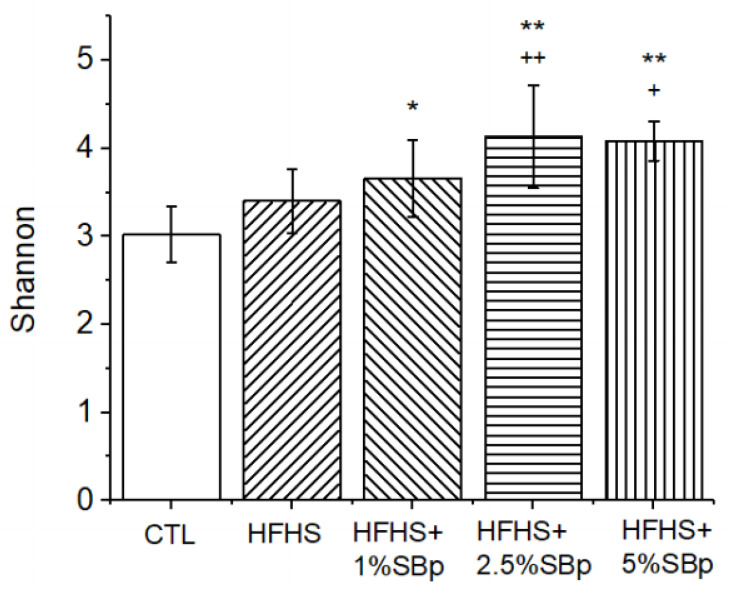
Effect of HFHS diet supplemented with SBp on α-diversity of gut microbiota in mice. Male C57 BL/J6 mice fed with low fat diet (control or CTL), HFHS diets or HFHS diet supplemented with 1%, 2.5% or 5% SBp for 11 weeks. Fecal samples were collected from individually caged mice at in the final weeks after of the dietary intervention. Shannon indices were expressed in mean (*n* = 8). *,**: *p* < 0.05 or 0.01 vs. CTL; +, ++: *p* < 0.05 or 0.01 vs. HFHS diet.

Supplementation with SBp (1–5%) to HFHS diet, but not HFHS diet alone, significantly increased Shannon index, a common indicator for α-diversity in gut microbiota, compared with that in mice receiving low fat diet (control) (*p* < 0.05 or 0.01). The levels of Shannon index in feces of mice treated with HFHS + 2.5% or 5% SBp, but not with 1% SBp, were significantly higher than in mice fed with HFHS diet alone (Figure 4).

**Figure 5:**
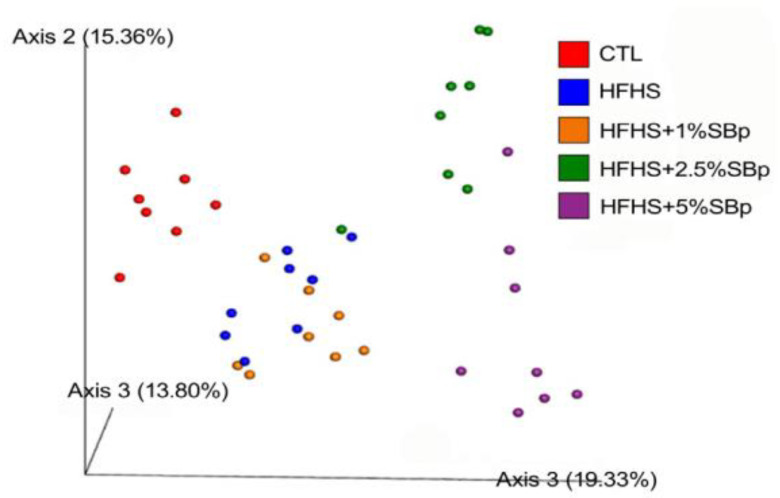
Effect of HFHS diet supplemented with SBp on ß-diversity of gut microbiota in mice. The experimental regimen is described in in the legend of Figure 4. Principle component analysis was based on Bray–Curtis dissimilarities between all sample sets (weighted by taxon abundance).

PCA analysis demonstrated that β-diversity in (control) mice fed a low fat diet completely separated from that of mice fed HFHS diet. β-Diversity in mice fed HFHS diet supplemented with 2.5% or 5% SBp completely separated from those receiving HFHS diet alone; however, in mice fed HFHS diet supplemented with 1% SBp, this partially overlapped with that in mice fed HFHS diet alone (Figure 5). 

### 3.4. Effect of Supplementation with Various Dosages of SBp on Phylum Bacteria in Mice

**Figure 6:**
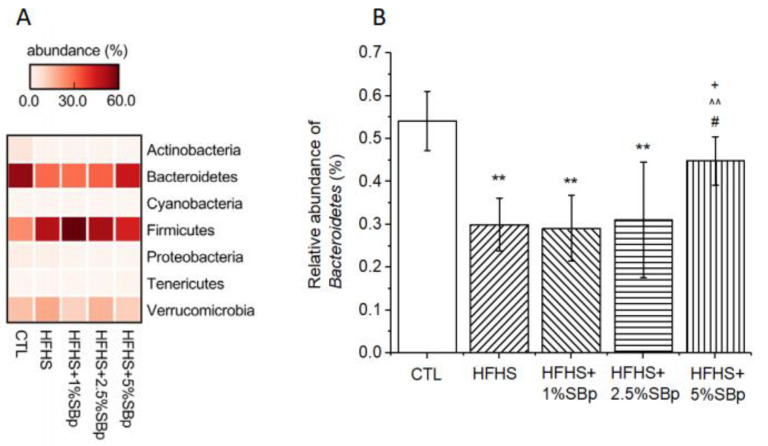
Effects of HFHS diet supplemented with or without SBp on the relative abundance of phylum bacteria and *Bacteroidetes* in feces of mice. The experimental regimen is described in the legend of Figure 4. (**A**) heat map of phylum bacteria; (**B**) relative abundances of *Bacteroidetes*. Values are expressed in median and range of *Bacteroidetes* (*n* = 8/group). **: *p* < 0.01 vs. the control (CTL) group; + *p* < 0.01 vs. the HFHS group; ^^: *p* < 0.01 vs. 1% SBp; #: *p* < 0.05 vs. 2.5% SBp.

The dominant phylum bacteria in the feces of mice fed various diets are *Bacteroidetes* and *Firmicutes* (Figure 6A). The relative abundances of *Bacteroidetes* in the control group mice receiving low fat diet was significantly higher than that in mice treated with HFHS diet (*p* < 0.01). In the group of mice treated with 5% SBp, the abundance of *Bacteroidetes* were significantly higher than that in mice receiving HFHS diet or HFHS + 1% SBp (*p* < 0.05, Figure 6B). The abundances of other phylum bacteria were not significantly different between groups.

### 3.5. Effect of Supplementation with Various Dosages of SBp on Family Bacteria in Mice

**Figure 7:**
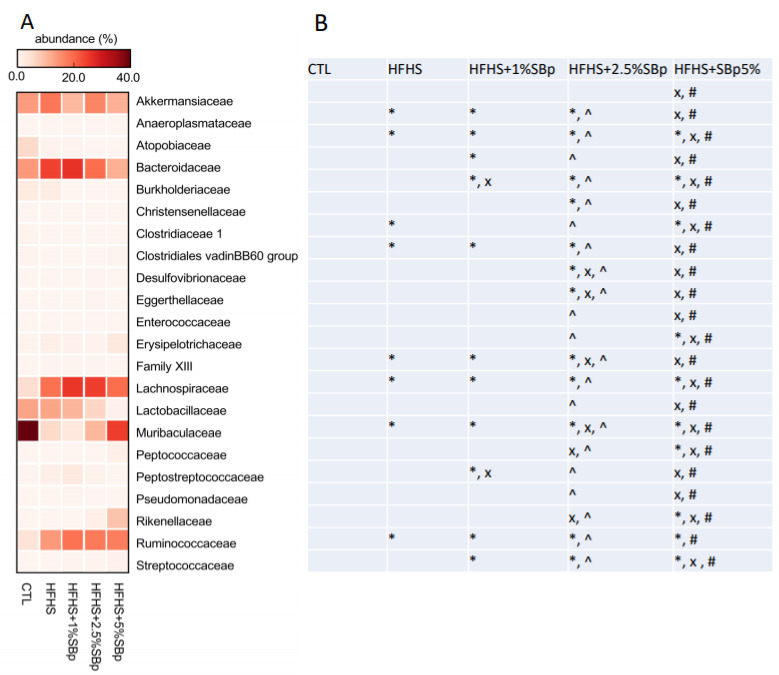
Effect of supplementation of SBp on the relative abundance of gut family bacteria. The experimental regimen was described in the legend of Figure 4. (**A**) Heat map of family bacteria; (**B**) statistical differences among mice with different diets (ANOVA and post-hoc Tukey test). *: *p* < 0.05 vs. control (CRL); x *p* < 0.05 vs. HFHS group; ^: *p* < 0.05 vs. 1% SBp; #: *p* < 0.05 vs. 2.5% SBp.

The major family bacteria in feces of mice fed with low fat, HFHS diet with or without SBp were *Akkemansiaceae, Bacteriodaceae, Lachnospiraceae, Lactobacillaceae, Muribaculaceae* and *Ruminococcaceae* (Figure 7A). The addition of SBp dose-dependently increased the abundance of *Muribaculaceae*, and decreased the abundances of *Akkemansiaceae, Bacteriodaceae* and *Lactobacillaceae* compared with HFHS diet alone (*p* < 0.05, Figure 7B). 

### 3.6. Correlation between Family Bacteria, Body Weights and Biochemical Variables

**Figure 8:**
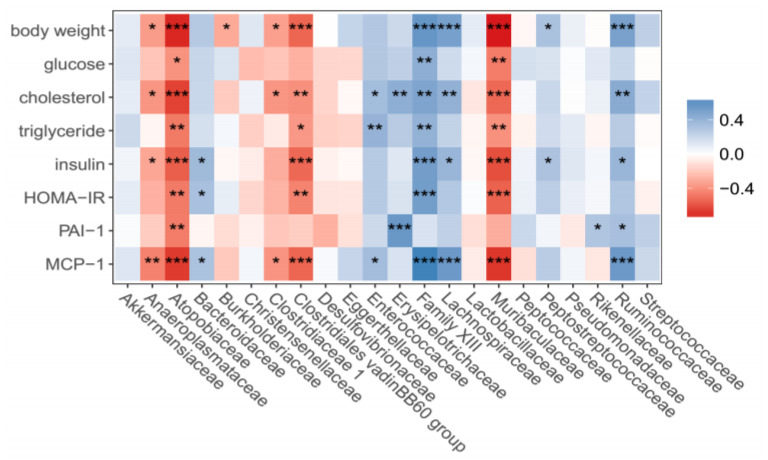
Correlation heat map of relative abundance of gut microbiota, at the family level, with physiological and biochemical parameters. The experimental regimen is described in the legend of Figure 4. *, **, ***: *p* < 0.05 or 0.01 or 0.001 in positive (blue) or negative (red) correlations between the abundance of each gut family bacteria and physiological or biochemical variables in the mice (*n* = 40).

The abundance of *Muribaculaceae, Atopobiaceae* and *Clostridiales vadinoBB60 group* family bacteria negatively correlated with body weights, cholesterol, triglycerides, lipids, insulin, HOMA-IR, MCP-1 or PAI-1 in mouse peripheral circulation (*p* < 0.05–0.001). The abundances of *Family XIII, Lachnospiraceae* and *Ruminococcaceae* family bacteria positively correlated with body weights, lipids, insulin or MCP-1 or PAI-1 in the plasma of mice (*p* < 0.05–0.001, Figure 8). 

## 4. Discussion

The major novel findings generated from the present study include: (i) oral administration of 1–5% SBp significantly reduced HFHS diet-induced hyperglycemia, hypercholesterolemia, hypertriglyceridemia and insulin resistance, but did not significantly alter body weight, in mice compared with those fed HFHS diet alone; (ii) supplementation of 1–5% SBp significantly attenuated HFHS diet-induced elevations in inflammatory markers in plasma; (iii) the administration of 2.5% or 5% SBp supplement in HFHS diet resulted in a complete separation of the β-diversity of gut microbiota from that in mice treated with HFHS diet alone, while that of mice receiving 1% SBp partially overlapped the results for HFHS diet-fed mice; (iv) SBp supplementation to HFHS diet dose-dependently increased the abundances of fecal *Bacteroidetes* phylum and *Muribaculaceae* family bacteria in mice compared with that of mice receiving HFHS diet alone. The abundances of *Muribaculaceae* negatively correlated with HFHS diet-induced metabolic and inflammatory markers in mice.

Previous studies on the hypoglycemic effects of SBp on HFHS or HFHS or high fat diet-induced hyperglycemia in mice or rats were only tested in one dose in mice (5% SBp) or rats (2.7% SBp) [13,14]. Dose-response of experimental animals to multiple dosages of oral administration of SBp on hyperglycemia is required to be determined for further pre-clinical and clinical studies. The results of the present study demonstrated that 1–5% SBp significantly reduced FPG, insulin, HOMA-IR, lipids and inflammatory markers in mice in a dose-dependent manner. The results of the present study suggest that 1% dried SB in mice or 15g/day of dried SB in humans (based on 1.5 kg or the average of daily food intake of humans) potentially achieves tested metabolic and anti-inflammatory benefits, and 2.5% dried SB in mice or 37.5 g/day of dried in humans may cause an evident improvement in gut microbiota, according to the present study, in mice. The results should be verified in future studies based on species. 

SBp supplementation in HFHS diet in the present and previous studies did not significantly alter body weight in mice [13,19,20]. Our recent experiments assessed the sizes of adipocytes in epidermal tissue in mice. HFHS diet increased the size of adipocytes, but the supplementation of SBp did not significantly affect the size of adipocytes compared to that in mice treated with HFHS diet alone [20]. However, du Preez et al. found that SBp treatment significantly reduced body weight and adipose deposition in rats compared to those fed with high fat diet alone [14]. The glucose-lowering and liver-protective effects were detected in both mice [20] and rats [14]. It is unclear whether the differential responding in body weight and fat deposition is due to the species difference or other factors between the experiments. 

SBp contains high levels of antioxidant polyphenols. The levels of anthocyanin in SBp were significantly higher than that in blue berry, strawberry or chokeberry [12]. Our previous study demonstrated that the predominant types of anthocyanin in SBp are cyanidin-3-galatoside (C3Ga, 74% *w/w*) and cyanidin-3-glucoside (C3G, 18% *w/w*) [15]. Previous studies demonstrated that C3G increased the translocation of glucose transporter-4 in skeletal muscle through the activation of insulin and AMP activated protein kinase pathway in mice [21]. C3G is 3-times more potent than C3Gs on the inhibition of endoplasmic reticulum stress in vascular endothelial cells induced by glycated low-density lipoprotein [22]. C3G supplementation decreased the levels of inflammatory mediators, PAI-1 and MCP-1, in plasma of mice fed HFHS diet [18]. The present study demonstrates that HFHS diet increased the levels of TNFα, PAI-1 and MCP-1 in mouse plasma compared to HFHS diet alone. Supplementation with 1–5% SBp in HFHS diet reduced the levels of inflammatory markers compared to mice receiving HFHS diet alone. The combination of the results supports the hypothesis that the anti-inflammatory and metabolic benefits of SBp in HFHS diet results from the amount of anthocyanin in SBp.

The results of PCA analysis indicated that the β-diversity of gut microbiota in mice receiving SBp is dose-dependent. The microbiota in feces of mice treated with HFHS diet plus 2.5% or 5% SBp was completely separated from that in mice fed with HFHS diet alone, but that in mice treated with 1% SBp was only partially separated from HFHS group. The findings suggest that SBp administration alters the β-diversity of gut microbiota in mice in a dose-dependent manner. Daily intake of 1% SBp moderately changes the diversity of microbiota in mouse gut. The results suggest that intake ≥2.5% of SBp is able to distinguishably alter gut taxonomy in mice. 

The effects of different dosages of SBp on phylum and family bacteria in feces of mice are consistent with our previous reports in general [14,18]. The results of the present study demonstrated that SBp administration dose-dependently increased the levels of *Bacteroidetes* phylum bacteria in feces of mice. Decreased abundance of *Bacteroidetes* in feces was detected in obese and diabetic animals and humans [23,24]. Positive correlation between SBp dosages and the abundances of *Muribaculaceae* family bacteria and negative correlation between the dosages of SBp and the relative abundances of *Lachonospiraceae* or *Bacteroidaceae* family bacteria were detected in the mice. *Muribaculaceae* family bacteria represents a large portion of *Bacteroidetes* phylum bacteria in rodents and has been detected in human gut microbiota [25]. A recent study demonstrated that the abundances of *Muribaculaceae* positively correlated with the levels of short chain fatty acids (SCFA) in mice [26]. SCFAs are generated from the digestion of intestinal fiber by a group of gut microbiota [27]. The production of SCFA in the feces of T2D patients was increased following the administration of fiber-rich diet and associated with the decrease in FPG and insulin resistance [28]. The combination of the findings suggests that SBp administration dose-dependently influenced multiple types of gut microbiota at phylum or family levels. Additional studies may further investigate the changes in gut microbiota in SBp treated animal models at the levels of genus, species and even strains for understanding the microbiotic mechanism for the prebiotic effect of SBp.

In addition to the negative correlation between *Muribaculaceae* family bacteria and body weights, metabolic or inflammatory variables, significant negative correlations between *Atopobiaceae*, a family bacteria of *Coriobacteriia* class in *Actinobacteria* phylum bacteria, and the physical or biochemical variables related to diabetes were detected in the present study. A recent study reported that canola meal increased abundance of *Atopobiaceae* in gut microbiota in chicken. The increase in the abundance of *Atopobiaceae* induced by canola meal was associated with the increases of the abundances of SCFA in cecal digesta in chicken [29]. The abundance of *Atopobiaceae* was evidently higher in the mice received low fat diet than that in mice receiving HFHS with or without SBp in the present study, which suggest that metabolic benefits of SB in HFHS diet-fed mice may not be directly related to the changes in *Atopobiaceae*. The present study also demonstrated that the abundances of *Lachnospiraceae, Ruminococcaceae* and *Family XIII* family bacteria positively correlated with body weights, glucose, lipids and inflammatory markers in mice, which are consistent with the results from previous studies on those bacteria [30,31,32]. 

Limitations of the present study include: (1) the health benefits of different dosages of SB were only available in mice. The results from the present study will need to be confirmed in other animal models and humans; (2) the animal experiments for dietary intake of SB was only conducted in male mice or rats. The impact of SB on female and pregnant animals have not be tested; (3) anthocyanins, including C3G and C3Ga, are strong candidates for the functional compounds of SB for its health benefits; however, the contribution of other components in SBp to the health benefits of this natural product has not been excluded. 

In conclusion, the results of the present study show that oral administration of SBp (≥1% mass of daily dry foods) resulted in significant metabolic and inflammatory benefits in mice fed HFHS diet. Distinct changes in the diversity of gut microbiota were detected in mice receiving ≥2.5% SBp, and the changes in gut microbiota correlated with metabolic and inflammatory variables. The combination of the findings suggests that oral administration of SBp reduces contemporary patterns of diet-induced hyperglycemia, hyperlipidemia, chronic inflammation and gut dysbiosis in a dose-dependent pattern. The results generated from animal experiments are required to be verified in clinical trials before use in diabetic patients.

## Figures and Tables

**Table 1:** Difference in body weight, metabolic and inflammatory variables between mice received low fat diet and high fat-high sucrose (HFHS) diet. HOMA-IR: homeostatic model assessment-insulin resistance; PAI-1: plasminogen activator inhibitor-1; TNFα: tumor necrosis factor-α; MCP-1: monocyte chemotactic protein-1. Values were expressed in mean ± SD (*n* = 8/group). **: *p* < 0.01 vs. low fat diet group.

Variables	Low Fat Diet	HFHS Diet
Body weight (g)	26.3 ± 0.99	41.94 ± 6.53 **
Glucose (mg/dL)	79.28 ± 4.58	123.65 ± 5.16 **
Cholesterol (mg/dL)	83.87 ± 5.60	126.06 ± 6.04 **
Triglyceride (mg/dL)	80.17 ± 6.55	125.74 ± 6.70 **
Insulin (ng/mL)	1.07 ± 0.14	2.32 ± 0.23 **
HOMA-IR	3.78 ± 0.52	12.75 ± 1.23 **
PAI-1 (ng/mL)	18.51 ± 5.33	42.58 ± 6.63 **
TNFα (pg/mL)	150.51 ± 16.51	308.51 ± 21.52 **
MCP-1 (pg/mL)	117.79 ± 18.44	237.62 ± 30.29 **

## Data Availability

The datasets analyzed during the current study will be available from the corresponding author on reasonable request.
